# Myeloid-specific TAK1 deletion results in reduced brain monocyte infiltration and improved outcomes after stroke

**DOI:** 10.1186/s12974-018-1188-3

**Published:** 2018-05-17

**Authors:** Anjali Chauhan, Jacob Hudobenko, Abdullah Al Mamun, Edward C. Koellhoffer, Anthony Patrizz, Rodney M. Ritzel, Bhanu P. Ganesh, Louise D. McCullough

**Affiliations:** 1 Department of Neurology, University of Texas McGovern Medical School at Houston, Houston, TX 77030 USA; 2 Department of Anesthesiology and Center for Shock, Trauma and Anesthesiology Research (STAR), University of Maryland School of Medicine, Baltimore, MD 21201 USA; 3 Memorial Hermann Hospital-Texas Medical Center, Houston, TX 77030 USA

**Keywords:** Ischemic stroke, Monocytes, Neutrophils, Inflammation, TAK1

## Abstract

**Background:**

Activation of transforming growth factor-β-activated kinase 1 (TAK1) occurs after stroke and leads to an exacerbation of brain injury. TAK1 is involved in innate and adaptive immune responses, but it has divergent inflammatory effects that are dependent on the cell type in which it is activated. There is a robust infiltration of myeloid cells after stroke; however, the contribution of myeloid TAK1 to cerebral ischemia is currently unknown. We hypothesized that myeloid-specific deletion of TAK1 would protect against ischemic brain injury.

**Methods:**

Myeloid TAK1^Δ^M and wild-type (WT) mice were subjected to middle cerebral artery occlusion (MCAo). Brain-infiltrating and splenic immune cells were evaluated at 3 days after stroke. Assessment of infarct size and behavioral deficits were performed on days 3 and 7 post-stroke.

**Results:**

Infarcts were significantly smaller in TAK1^Δ^M mice (*p* < 0.01), and behavioral deficits were less severe despite equivalent reduction in cerebral blood flow. Flow cytometry demonstrated an increase in the frequency of splenic monocytes and neutrophils (*p* < 0.05) and a decrease in splenic CD3^+^ T (*p* < 0.01) and CD19^+^ B (*p* = 0.06) cells in TAK1^Δ^M mice compared to WT at baseline. Three days after stroke, a significant increase in the number of brain-infiltrating immune cell was observed in both TAK1^Δ^M (*p* < 0.05) and WT (*p* < 0.001) mice compared to their respective shams. However, there was a significant decrease in the infiltrating CD45^hi^ immune cell counts (*p* < 0.05), with a pronounced reduction in infiltrating monocytes (*p* < 0.001) in TAK1^Δ^M after stroke compared to WT stroke mice. Additionally, a significant reduction in CD49d^+^ monocytes was seen in the brains of TAK1^Δ^M stroke mice compared to wild-type mice. Importantly, TAK1^Δ^M MCAo mice had smaller infarcts and improved behavioral outcomes at day 7 post-stroke.

**Conclusion:**

Our results showed that deletion of myeloid TAK1 resulted in smaller infarcts and improved functional outcomes at the peak of inflammation (day 3) and a reduction in brain-infiltrating immune cells that were primarily monocytes. Myeloid TAK1 deletion was also protective at 7 days post MCAo, reflecting a detrimental role of myeloid TAK1 in the progression of ischemic injury.

**Electronic supplementary material:**

The online version of this article (10.1186/s12974-018-1188-3) contains supplementary material, which is available to authorized users.

## Background

Transforming growth factor (TGF)-β-activated kinase 1 (TAK1) is a MAP3 kinase, upstream of several pathways that are involved in cerebral ischemic injury [1, 2]. TAK1 is activated by TGF-β, interleukin-1β, tumor necrosis factor-α, and other cytokines secreted after ischemic injury [3, 4]. Numerous studies have shown that site-specific ubiquitination and phosphorylation of TAK1 results in its activation [5–7]. TAK1 activation, in turn, induces the production of pro-inflammatory cytokines, chemokines, and activates immune cells that are known to play a detrimental role in stroke pathology [8, 9]. TAK1 is an essential gene and global deletion of TAK1 is embryonically lethal [10]. Growing evidence has shown that TAK1 is activated in neonatal hypoxia-ischemia, in in vitro models of oxygen-glucose deprivation, and after middle cerebral artery occlusion (MCAo), where it was demonstrated to aggravate injury [1, 2, 11]. Pharmacological inhibition of TAK1 reduces ischemic damage in a transient focal ischemia model [1, 2]. TAK1 is essential for protection against cytokine-induced cell death [12] and exerts anti-necrotic and antitumor activity [13]. Conditional tissue and hematopoietic-specific TAK1 ablation resulted in spontaneous tissue death [14–16] and apoptosis in a variety of cell types [17–19] but a recent study in which TAK1 was deleted from the alveolar macrophages and lung fibroblasts demonstrated attenuation of both inflammation and fibrosis in an experimental model of pneumoconiosis [20]. Thus, it is increasingly recognized that TAK1 regulation and function occurs in a tissue- and cell-specific manner.

There is ample evidence that the inflammatory response to stroke contributes to injury [21–23]. After ischemic stroke, there is an increase in monocyte and neutrophil release from the bone marrow and spleen [24, 25]. Stroke leads to blood-brain barrier compromise, microglial activation, and invasion of peripheral immune cells into the brain [26, 27]. Monocytes and neutrophils traffic to the brain secondary to increased expression of adhesion molecules, cytokines/chemokines, proteases, and reactive oxygen species production [28, 29]. TAK1 is ubiquitously expressed on many cell types including macrophages, neutrophils [17, 30–32], T cells [18, 33], B cells [19, 34], epidermal [14, 15], and intestinal epithelial cells [35]. Microglial-specific deletion of TAK1 reduced disease severity and immune cell infiltration in an experimental autoimmune encephalomyelitis model by inhibiting NFκB, JNK, and ERK1/2 signaling and dampening the inflammatory immune responses [36], suggesting a detrimental role of TAK1 in CNS inflammation. Hence, inhibition of TAK1 activation could be a potential neuroprotective strategy. However, a fundamental question remains: is myeloid TAK1 a key regulator of brain inflammation and its subsequent inactivation beneficial in the ischemic stroke? We hypothesized that myeloid TAK1 is a major contributor to cerebral ischemic damage as these cells aggressively infiltrate the brain and are involved in the evolution of brain injury. Conditional deletion of TAK1 in myeloid cells resulted in significant reduction of infiltrating monocytes into the brain and improved functional and infarct outcomes after ischemic stroke in mice.

## Methods

### Animals

TAK1^fl/fl^ mice (a gift from Dr. Wang at University of Texas MD Anderson Cancer Center) were crossed with lysozyme M-Cre (*LyzMCre*) mice (Jackson Laboratory) to obtain TAK1^Δ^M mice in the C57BL/6 background. Young adult male mice (7–8 weeks) of age were group housed in the pathogen-free housing and had access to food and water ad libitum. Our pilot experiments showed no difference in the splenic immune cell composition between TAK1^fl/+^ LysM ^cre/+^ and wild-type (WT) sham and MCAo cohorts. Thus, we used WT littermates as an experimental control group for further studies. All procedures were performed in accordance with NIH guidelines for the care and use of laboratory animals and were approved by the Institutional Animal care and use committee of the University of Texas Health Science Center.

### Transient stroke model

Transient cerebral ischemia was induced by 90 min of reversible middle cerebral artery occlusion (MCAO as previously described [37]. Rectal temperatures were maintained at approximately 37 °C during surgery and ischemia with an automated temperature control feedback system. A midline ventral neck incision was made, and unilateral MCAO was performed by inserting a 6.0 Doccol monofilament (Doccol Corp, Redlands, CA, USA) into the right internal carotid. Cerebral blood flow (CBF) was measured by Laser Doppler flowmetry (Moor Instruments Ltd., Devor, England). CBF was measured before ischemia, during ischemia, and at the time of reperfusion. Following reperfusion, mice were sacrificed at day 3 after stroke. Sham controls were subjected to same procedure except the suture was not introduced into the internal carotid artery. Animals were randomly assigned into the stroke and sham surgery groups. For the 7-day outcome studies, mice were subjected to 60 min of ischemia followed by reperfusion.

### Functional testing

Neurological deficit scores (NDS) were assessed at 3 and 7 days after a stroke on a four-point scale as described previously [38]. Corner testing (sensorimotor) and open field were performed at 3 and 7 days after stroke as described previously [39].

### Tissue harvesting

Mice were euthanized day 3 post-ischemia. Mice were transcardially perfused with 60 mL of cold, sterile PBS. The olfactory bulb, brainstem, and cerebellum were removed. The brain was then divided along the interhemispheric fissure into two hemispheres and subsequently rinsed with PBS to remove adherent cells.

### Flow cytometry

Spleens were removed and processed by mechanical disruption on a 70 μm filter screen. Red blood cell lysis was achieved by three consecutive 5–10 min incubations with Tris-ammonium chloride (Stem Cell Technologies). The ipsilateral hemisphere was placed in RPMI (Lonza) medium and mechanically and enzymatically digested in collagenase/dispase (1 mg/mL) and DNAse (10 mg/mL; both Roche Diagnostics). The cell suspension was filtered through a 70 μm mesh filter. Leukocytes were harvested from the interphase of a 70%/30% Percoll gradient. Spleen and brain leukocytes were washed with 1X PBS and blocked with mouse Fc Block (eBioscience, 1 μl/50 μl) prior to staining with primary antibody-conjugated fluorophores (CD45-AF700, CD11b-APC-eF780, Ly6G-FITC, Ly6C-APC, and CD49D-PE were purchased from eBioscience). For live/dead discrimination, a fixable viability dye was used (THERMOFISCHER SCIENTIFIC). Data were acquired on CytoFLEX cytometer (BECKMAN COULTER) and analyzed by FlowJo (TREESTAR INC.). Cell-specific fluorescence minus one controls were used to confirm individual antibody specificity. In spleen, monocytes and neutrophils were identified as (CD45^+^CD11b^+^Ly6C^+^ Ly6G^−^) and (CD45^+^CD11b^+^Ly6C^−^ Ly6G^+^) respectively. T cells were identified by (CD45^+^CD11b^−^CD3^+^) and B cells by (CD45^+^CD11b^−^CD19^+^). In the brain, peripheral monocytes (Ly6C^+^), neutrophils (Ly6G^+^) were gated on CD45^hi^ CD11b^+^, whereas peripheral lymphoid cells were gated on CD45^hi^ CD11b^−^ population Additional file 1.

### TTC and cresyl violet staining

Two cohorts of mice were examined that were euthanized at day 3 and 7 post-ischemia. For TTC staining, the animals were euthanized; brains were harvested and stored at − 80 °C for 4 min to slightly harden the tissue. Five, 2 mm coronal sections were cut from the olfactory bulb to the cerebellum and then stained with 1.5% TTC (SIGMA, St. Louise, MO). Slices were formalin-fixed (4%) and then digitalized for assessing infarct area using Sigma Scan Pro software as previously described [40]. The final infarct area is presented as percentage area (percentage of contralateral structures with correction for edema). For cresyl violet (CV) staining, animals were euthanized on day 7 after ischemia as described previously [41]. The infarct area of each brain was measured by an investigator blinded to the surgical groups, using image analysis software (Sigmascan Pro 5).

Naïve wild-type and TAK1^Δ^M (*n* = 3) mice were anesthetized and perfused with cold sterile PBS followed by 4% paraformaldehyde. A volume of 2 mL India ink (Sigma) and ferrous sulfate (1% in 20% India ink in PBS) was injected through the left ventricle. The animals were decapitated and brains were harvested with a circle of intact Willis. The brains were placed in 10% formalin overnight at 4 °C and examined for large vessel anatomy.

### Statistical analysis

**Fig. 1:**
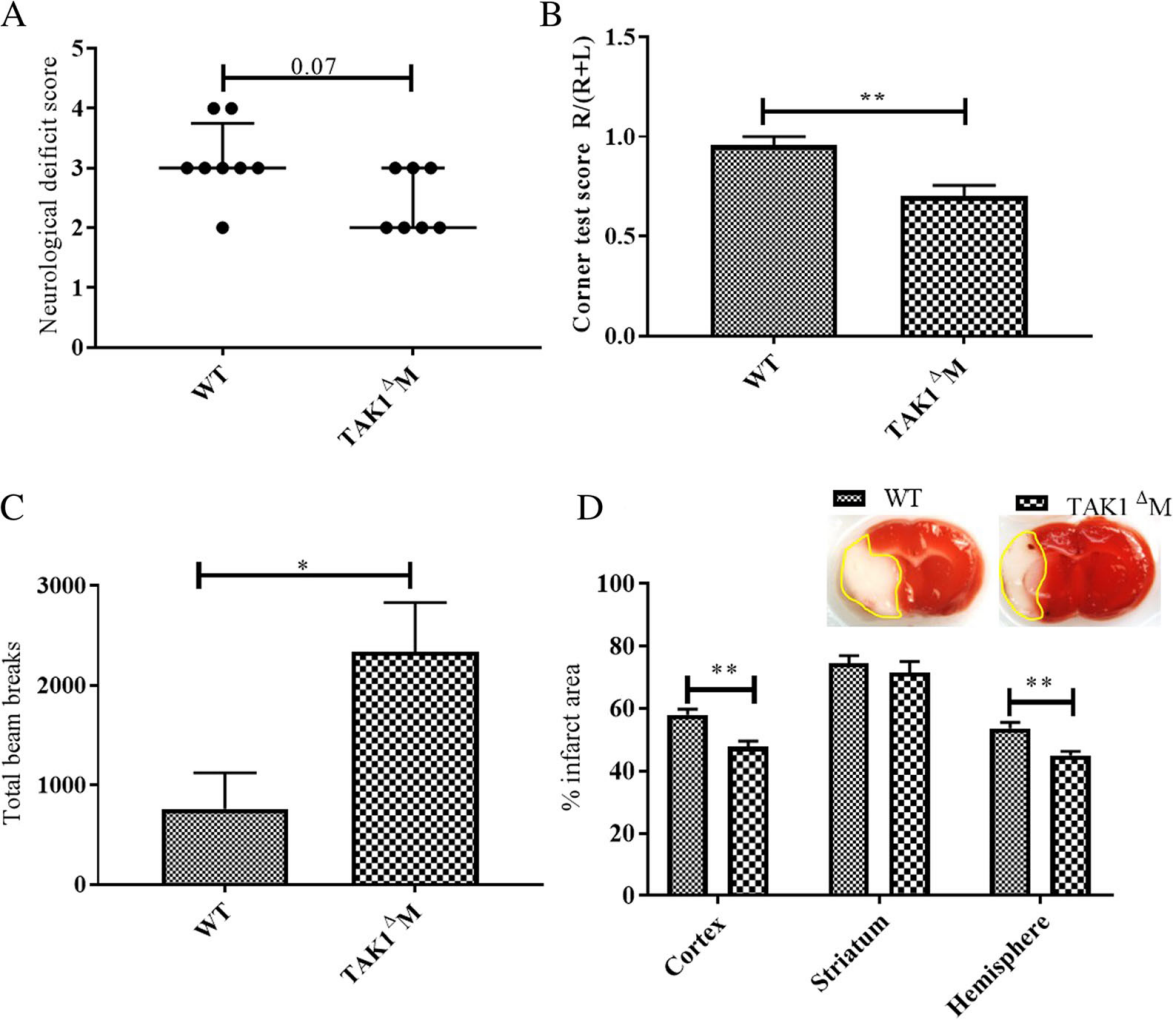
Deletion of TAK1 from myeloid cells resulted in reduced infarct and improved functional outcomes at day 3 post stroke. **a** TAK1^Δ^M mice had reduced neurological deficit scores (NDS). **b** Decreased turning bias on the corner test. **c** Increased locomotor activity. **d** Decreased infarct area at day 3 post stroke. Data is presented as Mean ± SEM, *n* = 6–8; *p < 0.05, **p < 0.01 (Two-sample *t* test or Wilcoxon rank-sum test)

**Fig. 2:**
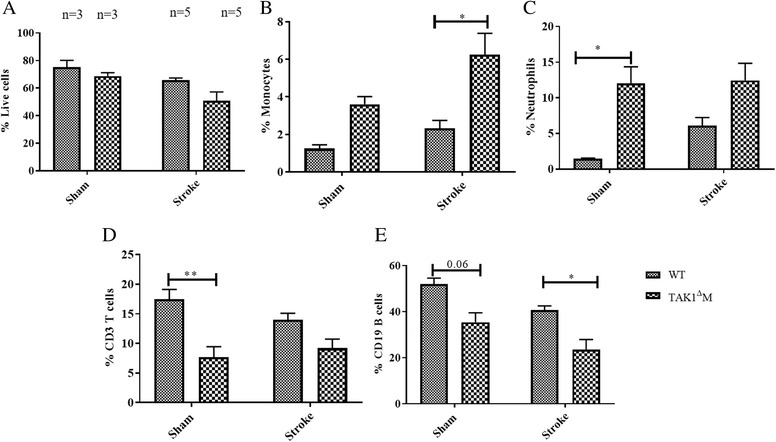
Increase in splenic monocytes and decrease in CD3^+^ T and CD19^+^ B cells in TAK1^Δ^M mice. **a** Frequency of live cells in sham and MCAo groups. **b** Increase in splenic monocytes. **c** Increase splenic neutrophils after deletion of TAK1 from myeloid cells. **d** Decline in splenic CD3^+^ T cells and **e** CD19^+^ B cells in TAK1^Δ^M mice. Data is presented as mean ± SEM, *n* = 3–5; *p < 0.05, **p < 0.01 (two-way ANOVA followed by post-hoc Tukey)

**Fig. 3:**
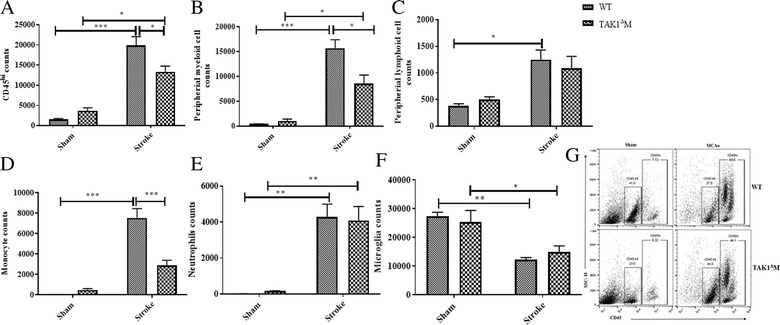
Decrease in brain-infiltrating immune cells in the TAK1^Δ^M mice after stroke. **a** Decrease in infiltrating CD45^hi^ immune cells. **b** Decline in infiltrating myeloid cells. **c** No difference in infiltrating lymphoid cells. **d** Decrease in monocyte counts. **e** No difference in the infiltrating neutrophils after stroke in TAK1^Δ^M mice MCAo mice at day 3. **f** No difference in microglia counts after stroke was seen in TAK1^Δ^M mice MCAo. **g** Representative flow plots showing infiltrating CD45^hi^ cells in the brain. Data is presented as mean ± SEM, *n* = 3–5; *p < 0.05, **p < 0.01, ***p < 0.001 (two-way ANOVA followed by post-hoc Tukey)

**Fig. 4:**
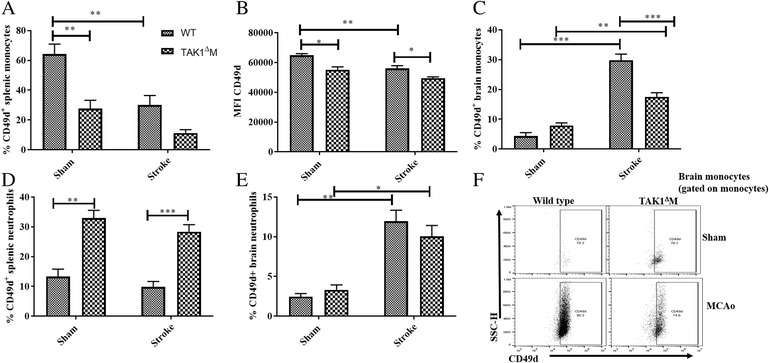
Decrease in splenic CD49d^+^ monocytes and decline in brain monocyte infiltration in TAK1^Δ^M mice after stroke. **a** Decrease in the frequency of CD49d^+^splenic monocytes. **b** Decrease in the CD49d MFI on splenic monocytes. **c** Decrease in frequency of CD49d^+^ monocyte in the brain of TAK1^Δ^M MCAo mice. **d** Increase in the frequency of splenic CD49d^+^ neutrophils. **e** No difference in the frequency of brain CD49d^+^ neutrophils after MCAo. **f** Representative flow plots showing CD49d gating on monocytes. Data is presented as mean ± SEM, *n* = 3–5; *p < 0.05, **p < 0.01, ***p < 0.001 (two-way ANOVA followed by post-hoc Tukey)

**Fig. 5:**
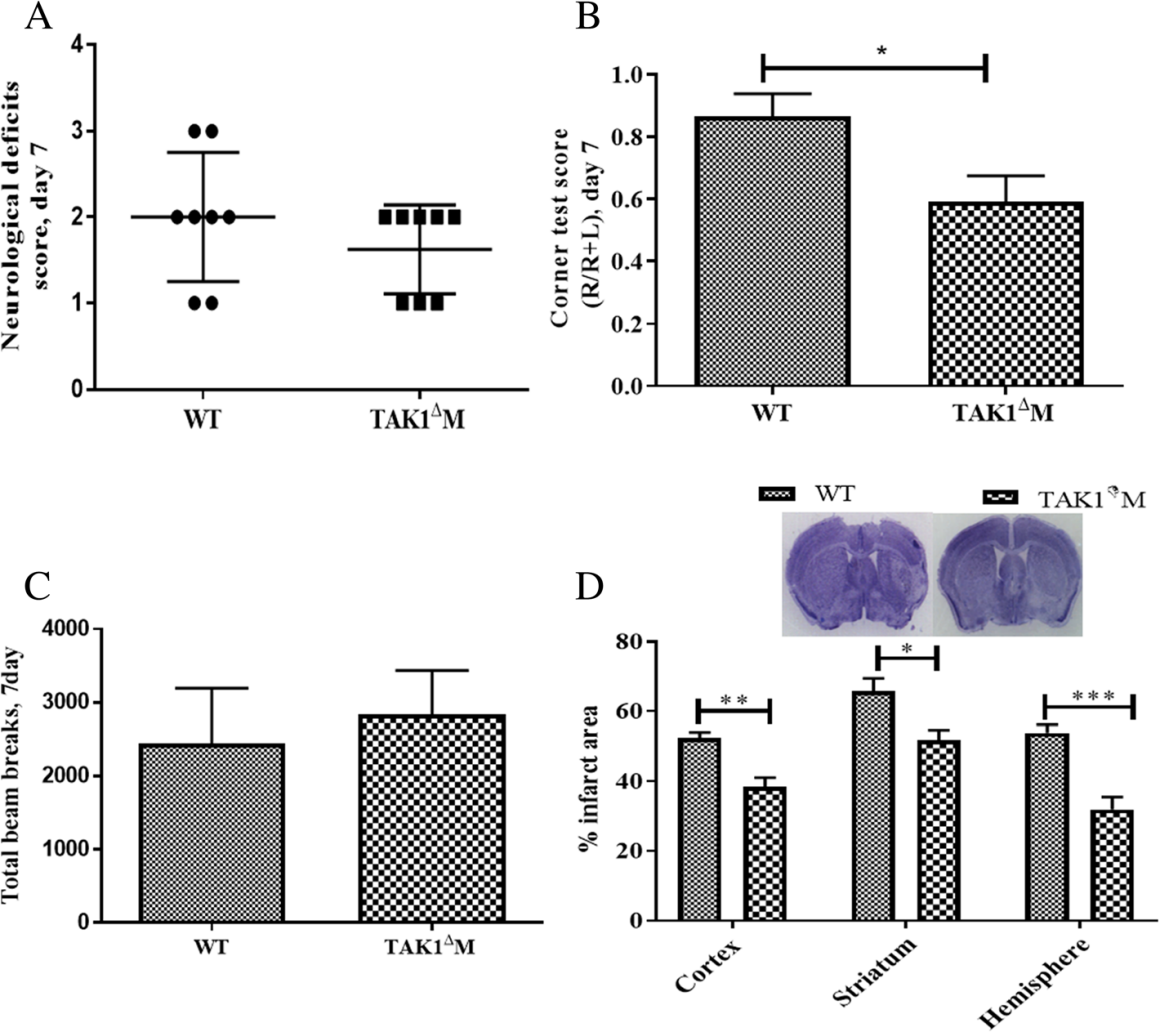
Deletion of TAK1 from myeloid cells resulted in reduced infarct and improved functional outcomes at day 7 post stroke. **a** TAK1^Δ^M mice had lower neurological deficit scores. **b** TAK1^Δ^M mice had less turning bias in the corner test. **c** No differences were seen in locomotor activity. **d** Decreased infarcts were seen at day 7 post stroke. Data is presented as mean ± SEM, *n* = 8; *p < 0.05, **p < 0.01 (two-sample *t* test or Wilcoxon rank-sum test)

Data is expressed as mean ± standard errors of mean (SEM) except for NDS, which was presented as median (interquartile range). A two-sample *t* test or Wilcoxon rank-sum test was used to compare variables between different groups (Figs. 1a–d and 5a–d). Two-way ANOVA followed by post-hoc test adjusted by Tukey method was used (Figs 2, 3, and 4). Statistical significance was set at *p* < 0.05. All statistical analyses were performed using GraphPad Prism 7.

## Results

### No difference in cerebral large vessel anatomy, cerebral blood flow, and body temperatures between WT and TAK1 ^Δ^M mice

No difference in the cerebral large vessel anatomy was observed between the WT and TAK1 ^Δ^M mice (Additional file 2: Figure S2: A). Additionally, there was no difference in the cerebral blood flow between WT and TAK1 ^Δ^M MCAo mice (Additional file 2: Figure S2: B). Furthermore, no difference in body temperature was observed between WT MCAo (36.50 ± 0.80) and TAK1 ^Δ^M MCAo (36.65 ± 0.93) mice.

### Deletion of myeloid TAK1 resulted in improved behavioral and infarct outcomes

To evaluate the role of myeloid TAK1 in cerebral ischemic injury, mice were subjected to 90 min of cerebral ischemia and behavioral assessments were done on day 3 post-stroke. The results of the Wilcoxon rank-sum test showed that the neurological deficit scores (NDS) were lower in the TAK1^Δ^M MCAo as compared to control TAK1 intact MCAo mice, though this did not reach statistical significance (*p* = 0.07, Fig. 1a). The Welch corrected *t* test results demonstrated a significant decline in the corner test scores in the TAK1 ^Δ^M MCAo mice compared to the control MCAo group (*p* = 0.0036, Fig. 1b). In the open field task, total beam breaks were higher in the TAK1^Δ^M MCAo vs control MCAo group reflecting an increase in locomotor activity (*p* = 0.023, Fig. 1c). A significant reduction in cortical infarct (*p* = 0.003) and hemispheric (*p* = 0.005) infarct was seen. There was no difference (*p* = 0.49) in the striatal infarct between both the MCAo groups. These results demonstrate that myeloid TAK1 deletion protects against stroke and reduces behavioral deficits.

### TAK1^Δ^M mice exhibited expansion of splenic myeloid populations and decreased lymphocytes

To investigate changes in the peripheral immune compartment, the spleen of the TAK1 ^Δ^M and control mice was immunophenotyped at day 3 post-stroke. Previous studies have shown that cell type and tissue-specific TAK1 deletion increases cell death in multiple cells and tissues including hematopoietic cells, monocytes, epidermis, and intestinal epithelium [14, 32, 35, 42].

We observed no difference in the frequency of splenic live cells between control and TAK1^Δ^M shams and MCAo groups (Fig. 2a). However, there was significant effect of myeloid TAK1 deletion on frequency of monocytes (*F* (1, 12) = 14.3, *p* = 0.0026) and neutrophils (*F* (1, 12) = 18.4, *p* = 0.0011). The splenic frequency of monocytes (Fig. 2b) and neutrophils (*p* = 0.024, Fig. 2c) was increased in the TAK1 ^Δ^M sham mice likely reflecting extramedullary hematopoiesis, which has also been reported [17]. Additionally, a significant effect of myeloid TAK1 deletion was observed on frequency of T cells (*F* (1, 12) = 22.1, *p* = 0.0005) and B cells (*F* (1, 12) = 21.6, *p* = 0.0006). Deletion of myeloid TAK1 resulted in a decrease in the splenic T (*p* = 0.008, Fig. 2d) and B cell frequencies (*p* = 0.06, Fig. 2e), hence we observed an expansion in splenic myeloid compartment and decrease in lymphocytes.

### Decrease in brain-infiltrating immune cells in TAK1^*Δ*^*M* mice after stroke

Stroke led to an increase in CD45^hi^ cells in the brain of both WT MCAo (*p* < 0.0001) and TAK1 ^Δ^M MCAo mice (*p* = 0.01) as compared to their respective sham controls. CD45^hi^ counts were significantly lower (*p* = 0.048) in the brains of TAK1^Δ^M MCAo as compared to WT MCAo mice (Fig. 3a). This was driven by a relatively selective decrease in myeloid infiltration after stroke as TAK1 ^Δ^M mice had significantly less infiltration of myeloid cells (*p* = 0.018, Fig. 3b) as compared to WT MCAo mice. This was primarily due to a significant reduction in the number of monocytes (Fig. 3d), but not neutrophils (Fig. 3e), reflecting a monocyte-specific mechanism. A stroke-induced decrease in microglia counts was evident in both the MCAo groups at day 3 post stroke (Fig. 3f). No difference was observed in the infiltration of peripheral lymphoid cells between TAK1^Δ^M MCAo as compared to WT MCAo mice, suggesting that the reduction in infiltrating monocytes was not simply reflective of the reduced infarct in TAK1^Δ^M mice (Fig. 3c).

### TAK1 loss in myeloid cells block monocyte extravasation into the brain

The spleen is highly innervated by the sympathetic nervous system. After a stroke, there is activation of a sympathetic nervous system that results in the involution of the spleen and transmigration of immune cells that correlates well with infarct size [43–45]. To infiltrate the brain, circulating leukocytes utilize the CD49d and VCAM axis, an important mediator of immune cell entry after injury [46–48]. A significant reduction (*p* = 0.006) in the frequency of splenic CD49d^+^ monocytes was seen in TAK1^Δ^M as compared to control sham mice Fig. 4a). Moreover, the mean fluorescence intensity (MFI) of CD49d on monocytes was significantly lower (*p* = 0.10) in the TAK1^Δ^M as compared to control sham mice (Fig. 4b). A significant decrease (*p* = 0.0044) in the frequency of splenic CD49d^+^ monocytes in the WT MCAo and TAK1^Δ^M MCAo (*p* = 0.210) was observed as compared to their respective shams (Fig. 4a). Additionally, a significant reduction on MFI was observed in WT MCAo (*p* = 0.009) as compared to WT sham. Moreover, TAK1^Δ^M MCAo has lower (*p* = 0.023) splenic CD49d MFI as compared to WT MCAo (Fig. 4b).

Subsequently, a stroke-driven increase in the brain CD49d^+^ monocyte numbers was observed in WT MCAo (*p* < 0.0001) and TAK1^Δ^M MCAo (0.022) as compared to their respective shams. Interestingly, TAK1^Δ^M MCAo had reduced frequency (*p* < 0.001) of CD49d^+^ monocytes as compared to WT MCAo mice. On the other hand, the frequency of splenic CD49d^+^ neutrophils was higher (*p* = 0.0013) in the TAK1 ^Δ^M as compared to control sham mice reflecting an increased extravasation of neutrophils in the spleens of these mice (Fig. 4d). Additionally, there was no difference in splenic CD49^+^ neutrophils in the TAK1^Δ^M MCAo (*p* = 0.56) and WT MCAo (*p* = 0.75) compared to their respective shams. A stroke-induced increase in CD49d^+^ neutrophil frequency was observed in the brains of both TAK1^Δ^M and WT MCAo mice (Fig. 4e). However, no difference in CD49d^+^ neutrophils was seen between TAK1^Δ^M and WT stroke animals. These results suggested that TAK1 loss in myeloid cells selectively affected monocytes transmigration to the brain after stroke, but did not affect the neutrophil migration.

### Smaller infarcts and improved functional outcomes in TAK1^Δ^M mice at day 7 post stroke

To evaluate functional outcomes and infarct size a week after injury, mice in both cohorts underwent a 60-min MCAo (Fig. 5). At day 7 after stroke, NDS and total beam breaks were not significantly different between the TAK1^Δ^M and control MCAo mice (*p* = 0.45, Fig. 5a, c), however, corner test scores were significantly lower (*p* = 0.033, Fig. 5b) in the TAK1^Δ^M MCAo as compared to WT MCAo mice, demonstrating a continued sensorimotor deficit in WT stroke mice that was ameliorated by myeloid TAK deletion. Additionally, TAK1^Δ^M MCAo mice had significantly smaller cortical (*p* = 0.002), striatal (*p* = 0.014), and hemispheric infarcts (*p* = 0.0008) as compared to WT MCAo animals (Fig. 5d).

## Discussion

**Fig. 6:**
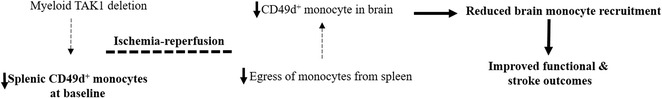
Proposed mechanism for reduced brain injury by myeloid TAK1 deletion. Loss of TAK1 in myeloid cells resulted in reduced CD49 integrin expression on splenic monocytes. After ischemia-reperfusion injury, attenuation of splenic monocytes egress from the spleen and recruitment of CD49d^+^ monocytes into the brain

This work demonstrates several novel findings which support our hypothesis that myeloid TAK1 is one of the key molecules essential for progression of cerebral injury in focal ischemic stroke. First, myeloid TAK1 knockout mice had smaller infarcts and improved functional outcomes at day 3 post-stroke. Secondly, myeloid TAK1^Δ^M mice had reduced infiltration of peripheral immune cells into the brain after stroke. Thirdly, there was a specific reduction in monocyte infiltration in the TAK1^Δ^M mice after stroke. Interestingly, there were fewer splenic CD49d^+^ monocytes in TAK1^Δ^M mice and a corresponding reduction in CD49d^+^ monocytes in the brains of TAK1^Δ^M mice after stroke that was associated with a reduction in injury (Fig. 6). Lastly, at day 7 post-stroke, TAK1^Δ^M MCAo mice had smaller infarcts and improved functional outcomes.

TAK1 is a member of a MAP3 kinase family and is activated by many diverse inflammatory stimuli. Once activated TAK1 and its adaptor proteins, TAB2, TAB3, and NEMO are recruited and phosphorylate TAK1 and activate κB kinase (IKK). IKK activation induces proinflammatory cytokines, chemokines, and activation of immune cells. Conversely, TAK1 protects cells against cytokine-induced death by producing anti-apoptotic proteins [49]. Additionally, TAK1 can confer anti-necrotic and anti-carcinogenic effects and helps maintain tissue homeostasis [17, 49]. The inflammatory response plays an essential role in stroke pathology. After a stroke, the breakdown of the blood-brain barrier results in activation of immune cells locally, the release of pro-inflammatory cytokines, and expression of adhesion molecules, which leads to the recruitment of immune cells from the circulation [50]. Stroke profoundly affects the peripheral immune system and inhibition of peripheral inflammation may prove to be an attractive strategy for stroke treatment. TAK1 is an important target, which is strategically placed upstream of many inflammatory signaling pathways including MAPK/Jnk, MAPK/Erk, NFκB. Pharmacological inhibition of TAK1 reduces infarct damage but may have off-target effects and inhibitors have difficulty crossing the blood-brain barrier, which would be less of a concern if the beneficial effects of TAK1 inhibition are secondary to suppression of peripheral immune cell TAK1 activation.

We examined mice with myeloid deletion of TAK1 to delineate the in vivo function of TAK1 after ischemic stroke. In order to produce a conditional deletion of TAK1 in myeloid cells, we used the Cre-LoxP system, in which Cre expression is under lysozyme M promoter. This mouse line has been extensively used to induce Cre expression in macrophages, neutrophils, and microglia [17, 32, 51–54]. Results from crossing LysM-Cre mice with reporter mice have shown 30–45% recombination in microglia [36, 55] and high recombination in peripheral monocytes and neutrophils. Animals with myeloid-specific TAK1 deletion were generated using the LysM-Cre mouse line. Deletion of TAK1 from neutrophils and monocyte/macrophages also microglia (Additional file 3: Figure S3), resulted in high circulating neutrophils, monocytes, and animals develop splenomegaly [17]. Tissue-specific deletion of TAK1 caused cell death in tissues including epidermis, intestinal epithelium, hepatocytes, and osteoblasts [32, 42]. Interestingly, myeloid-specific TAK1 deletion did not induce cell death in our study, a finding also seen by others [17, 30]. A previous study by Goldmann et al. [36] has shown that conditional deletion of TAK1 on microglia (using a cx3cr1 model) resulted in reduced autoimmune inflammation and axonal and myelin damage in experimental autoimmune encephalomyelitis model, demonstrating a role of microglial TAK1 in neuroinflammation. After a stroke, microglia are activated and release proinflammatory cytokines and leukocyte adhesion molecules. By binding to adhesion molecules, peripheral immune cells including neutrophils, monocytes, and lymphocytes are recruited to the site of injury. These infiltrated immune cells then release proinflammatory cytokines and further aggravate the cerebral injury [44, 45, 56]. TAK1 is important for myeloid cell function and is required for IKK and JNK activation which results in the production of pro-inflammatory cytokines [17]. After a stroke, the myeloid-specific TAK1 deleted animals had less infiltrated immune cells which were a consequence of reduced monocyte counts in these mice. No difference in the neutrophil counts after stroke was evident reflecting a monocytes-specific role. CD49d is expressed on the surface of all leukocytes including monocytes and neutrophils and is responsible for tethering, rolling, and firm adhesion to the blood vessels [57, 58]. Leukocytes use the CD49d and VCAM-1 axis to transmigrate to the site of brain injury [57, 58]. In the myeloid-specific TAK1 deleted mice, there were reduced CD49d^+^ monocytes reflecting less extravasation of peripheral monocytes after stroke. This decline in CD49d positivity was monocyte specific as no difference in CD49d^+^ neutrophils was observed after stroke in both the cohorts. Previous studies have shown that the monocytes from the myeloid TAK1-depleted animals have reduced stimulus-induced activation of IKK and JNK [17], which might suggest the reduced production of proinflammatory cytokines, though this needs to be further validated. Finally, we investigated whether the benefit of TAK1^Δ^M deletion was sustained at a second, later time point after the peak of immune cell infiltration. Both behavioral outcomes and infarct size were significantly better in TAK1^Δ^M MCAo animals reflecting that myeloid TAK1 deletion was still protective at day 7 post-ischemia.

Our findings suggest that myeloid TAK1 deletion results in neuroprotection, similar to what was seen with global pharmacological inhibition. However, this study has several limitations. First, we investigated the effect of myeloid TAK1 deletion at relatively acute time points after stroke, so the role of TAK1 in chronic repair and recovery remains to be explored. Secondly, we did not include female and older animals as this initial study was designed to explore if there was any role of myeloid TAK1 in acute stroke outcomes. Finally, as TAK1 was deleted from all myeloid cells, we cannot specifically delineate the role of central and peripheral TAK1 activation in stroke injury. Finally, constitutively deleting TAK1 from myeloid cells resulted in an increase in frequency of peripheral monocytes and neutrophil, though we did not evaluate the functional consequences of these changes, which could have affected aspects of monocyte and neutrophil physiology, hence the use of an inducible TAK1 deletion model would strengthen our findings and will be developed for use in future studies.

## Conclusions

In conclusion, we found that myeloid-specific TAK1 deletion reduced infarct injury and improved functional outcomes after stroke. The mechanism for the reduced cerebral damage in this group appears to be secondary to a specific and selective decrease in monocyte infiltration into the ischemic brain. Selective inhibition of TAK1 in peripheral myeloid cells could be a useful strategy for stroke therapy.

## Additional files

Additional file 1: **Figure S1.** Gating strategy for brain immune cells. (JPG 272 kb)

Additional file 2: **Figure S2.** Large vessel anatomy and cerebral blood flow changes A. No gross anatomical difference in the large blood vessels between WT and TAK1^Δ^M naïve mice (*n* = 3). B. No difference in cerebral blood flow between WT and TAK1^Δ^M MCAo mice (*n* = 5). (JPG 35 kb)

Additional file 3: **Figure S3.** mRNA expression of TAK1 in micro glia isolated from mouse brain. Microglia from the brain of WT and TAK1^Δ^M mice were isolated by cell sorting. Isolated cells were treated with RNA later. Extracted mRNA (3 μg) from the microglia population were converted to cDNA. The expression levels of the target genes were calculated with the relative standard curve method after normalizing the target gene expression to the expression of the house-keeping gene encoding glyceraldehyde 3-phosphate dehydrogenase (GAPDH). The expression of the latter gene was measured with the primers GAPDH-for (CAA GGT CAT CCA TGA CAA CTT TG) and GAPDH-rev (GTC CAC CAC CCT GTT GCT GTA G). Subsequently, the expression of the selected gene, TAK1, in the microglia of the experimental TAK1^Δ^M mice was corrected for physiological TAK1 expression levels in healthy WT mice which were housed under the very same conditions. The mRNA expression in the latter was determined as described above, the obtained values were set to 1.0 and used as the reference., Data is presented as mean ± SEM, *n* = 3–4 (Students *T* test). (JPG 23 kb)
