# Supplementary figures and images for: Myeloid-specific TAK1 deletion results in reduced brain monocyte infiltration and improved outcomes after stroke
Source: J Neuroinflammation. 2018 May 17;15:148. doi: 10.1186/s12974-018-1188-3 (PMC5960093; doi:10.1186/s12974-018-1188-3)

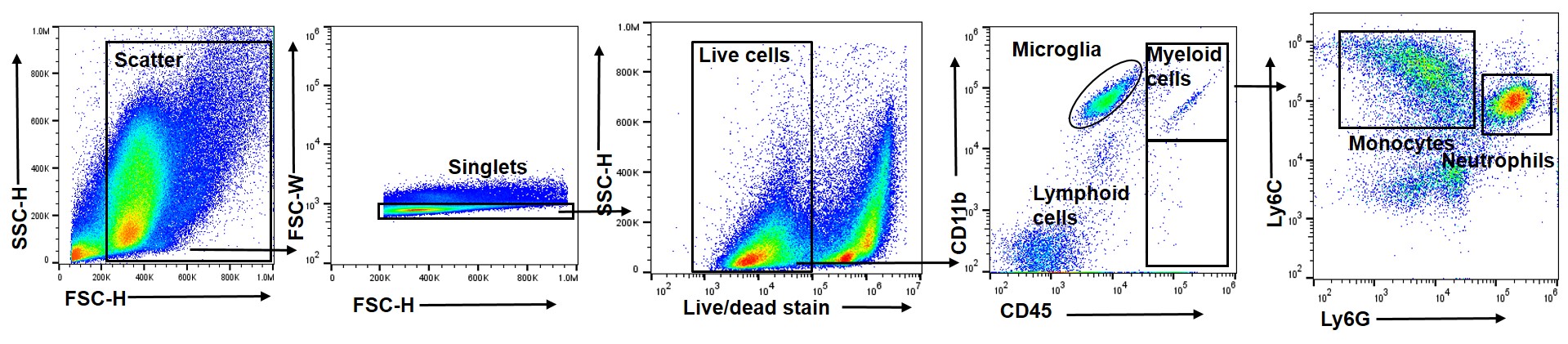

Supplement: Supplementary file 1 — Figure S1. Gating strategy for brain immune cells. (JPG 272 kb) [file 12974_2018_1188_MOESM1_ESM.jpg]

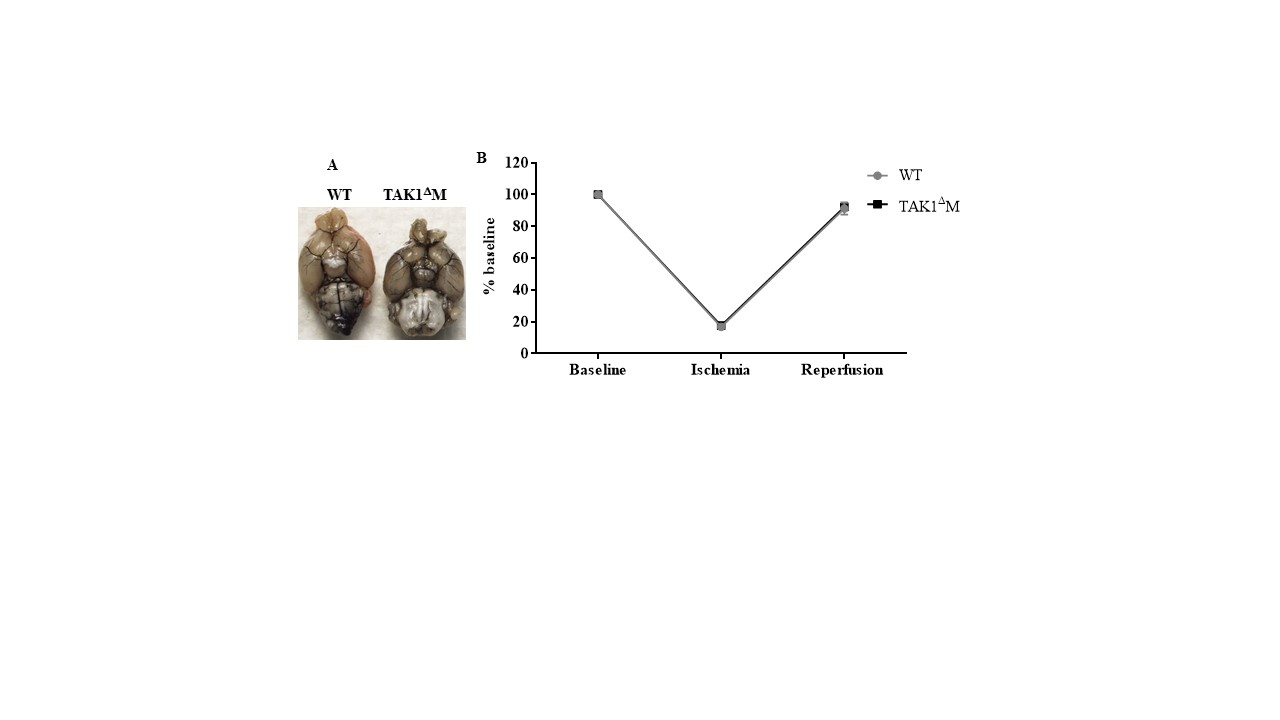

Supplement: Supplementary file 2 — Figure S2. Large vessel anatomy and cerebral blood flow changes A. No gross anatomical difference in the large blood vessels between WT and TAK1ΔM naïve mice (n = 3). B. No difference in cerebral blood flow between WT and TAK1ΔM MCAo mice (n = 5). (JPG 35 kb) [file 12974_2018_1188_MOESM2_ESM.jpg]

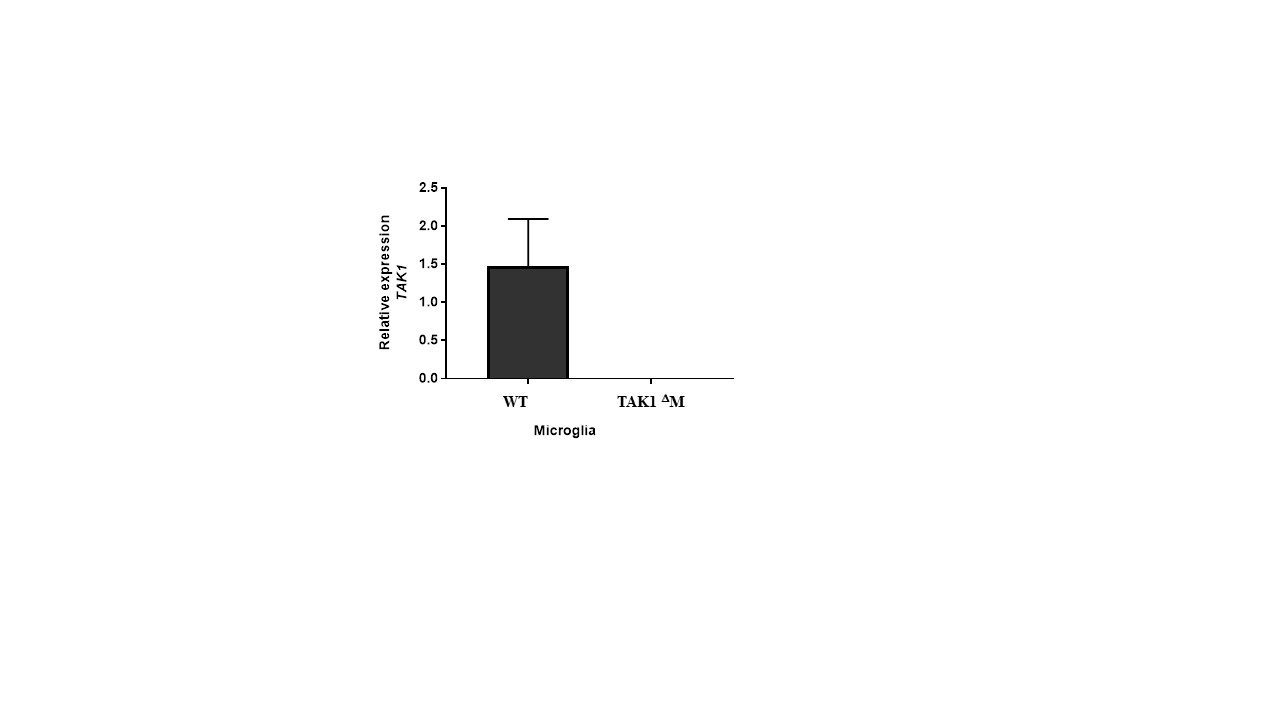

Supplement: Supplementary file 3 — Figure S3. mRNA expression of TAK1 in micro glia isolated from mouse brain. Microglia from the brain of WT and TAK1ΔM mice were isolated by cell sorting. Isolated cells were treated with RNA later. Extracted mRNA (3 μg) from the microglia population were converted to cDNA. The expression levels of the target genes were calculated with the relative standard curve method after normalizing the target gene expression to the expression of the house-keeping gene encoding glyceraldehyde 3-phosphate dehydrogenase (GAPDH). The expression of the latter gene was measured with the primers GAPDH-for (CAA GGT CAT CCA TGA CAA CTT TG) and GAPDH-rev (GTC CAC CAC CCT GTT GCT GTA G). Subsequently, the expression of the selected gene, TAK1, in the microglia of the experimental TAK1ΔM mice was corrected for physiological TAK1 expression levels in healthy WT mice which were housed under the very same conditions. The mRNA expression in the latter was determined as described above, the obtained values were set to 1.0 and used as the reference., Data is presented as mean ± SEM, n = 3–4 (Students T test). (JPG 23 kb) [file 12974_2018_1188_MOESM3_ESM.jpg]
